# Contrast-Induced Acute Kidney Injury in Patients on SGLT2 Inhibitors Undergoing Percutaneous Coronary Interventions: A Propensity-Matched Analysis

**DOI:** 10.3389/fcvm.2022.918167

**Published:** 2022-06-20

**Authors:** Rui Hua, Ning Ding, Hanqing Guo, Yue Wu, Zuyi Yuan, Ting Li

**Affiliations:** ^1^Department of Cardiovascular Medicine, The First Affiliated Hospital of Xi'an Jiaotong University, Xi'an, China; ^2^Department of Gastroenterology, Xi'an Central Hospital, Xi'an, China; ^3^Key Laboratory of Molecular Cardiology, Xi'an, China; ^4^Key Laboratory of Environment and Genes Related to Diseases, Ministry of Education, Xi'an, China

**Keywords:** SGLT2 inhibitor, contrast-induced AKI, percutaneous coronary intervention, coronary artery disease, diabetes mellitus

## Abstract

**Background:**

Contrast-induced acute kidney injury (CI-AKI) is a common complication of patients undergoing percutaneous coronary intervention (PCI). Data regarding the influence of sodium-glucose cotransporter-2 (SGLT2) inhibitor on the CI-AKI incidence and renal outcomes of patients undergoing PCI are limited. This study aimed to examine the real-world risk of CI-AKI in SGLT2 inhibitor users undergoing PCI.

**Methods:**

We used longitudinal data from the medical records of the First Affiliated Hospital of Xi'an Jiaotong University. We selected SGLT inhibitor users and nonusers [patients with type 2 diabetes (T2D) without SGLT2 inhibitor prescription] undergoing PCI. We determined CI-AKI by the ESUR (European Society of Urogenital Radiology, AKI_ESUR_) and KDIGO definition (Kidney Disease: Improving Global Outcomes, AKI_KDIGO_). We performed 1:1 nearest-neighbor propensity matching and calculated unadjusted odds ratios (ORs) and adjusted ORs (aORs; accounting for covariates poorly balanced) for AKI in primary and sensitivity analyses. We compared the renal function indicators in users and nonusers at 24, 48, and 72 h post-PCI.

**Results:**

We identified 242 SGLT2 inhibitor users and 242 nonusers in the cohort. The unadjusted ORs of CI-AKI_ESUR_ were 63% lower in users [OR: 0.37 (95% CI: 0.18–0.68); *P* = 0.01], which was unchanged [aOR: 0.37 (95% CI: 0.19–0.67); *P* < 0.01] post adjustment. These estimates did not qualitatively change across several sensitivity analyses. There was no significant difference in urea nitrogen, creatinine, and estimated glomerular filtration rate (eGFR) values between the two groups before PCI, and at 24 h, while the creatinine (48 and 72 h post-PCI) and CyC (24 and 48 h post-PCI) were significantly lower than those in the nonuser group (*P* < 0.05).

**Conclusion:**

Our findings do not suggest an increased risk of CI-AKI associated with SGLT2 inhibitor use in patients with CAD and T2D undergoing PCI.

## Introduction

Revascularization by the percutaneous coronary intervention (PCI) has achieved great success in reducing mortality for patients with coronary artery disease (CAD) (1). Nevertheless, a proportion of patients with CAD have suffered from an acute renal injury caused by contrast medium (CM) with the incidence ranging from 1.3 to 33.3% (2), which is defined as contrast-induced acute kidney injury (CI-AKI), considered as a new-onset or an exacerbation of renal dysfunction following administration of CM, without other potential causes. CI-AKI is an increasing complication in the general population of patients with CAD and the risk of this serious adverse event is further increased among patients with type 2 diabetes mellitus (T2DM) (3).

Sodium-glucose cotransporter 2 (SGLT2) inhibitors, which specifically inhibit renal tubular reabsorption of glucose, are new medications for the treatment of patients with T2DM. Mechanically, SGLT2 inhibitors (SGLT2i) block the reabsorption of glucose in the kidney, increase glucose excretion, and lower blood glucose levels (4). Several multicenter studies demonstrated lower rates of cardiovascular events and mortality, and a significant reduction in incidence and worsening kidney disease (5–7). However, little is known about the impact of SGLT2i on the incidence of CI-AKI for patients undergoing PCI.

Recently, real-world studies suggest that patients using SGLT2i, including empagliflozin, were at a lower risk of developing AKI and exhibited a smaller eGFR decline than patients using other glucose-lowering drugs (8). This indicates the potential renoprotective effects of SGLT2i against AKI for patients with type 2 diabetes. Few clinical evidence was reported to evaluate the impact of SGLT2i on the incidence of CI-AKI. In our study, we hypothesized that SGLT2i protect the renal function of patients with CAD and reduce CI-AKI incidence among patients undergoing PCI. Our study aims to explore the specific effect of SGLT2i on the incidence of CI-AKI and renal function among patients with CAD undergoing PCI.

## Materials and Methods

### Study Cohort

**Figure 1:**
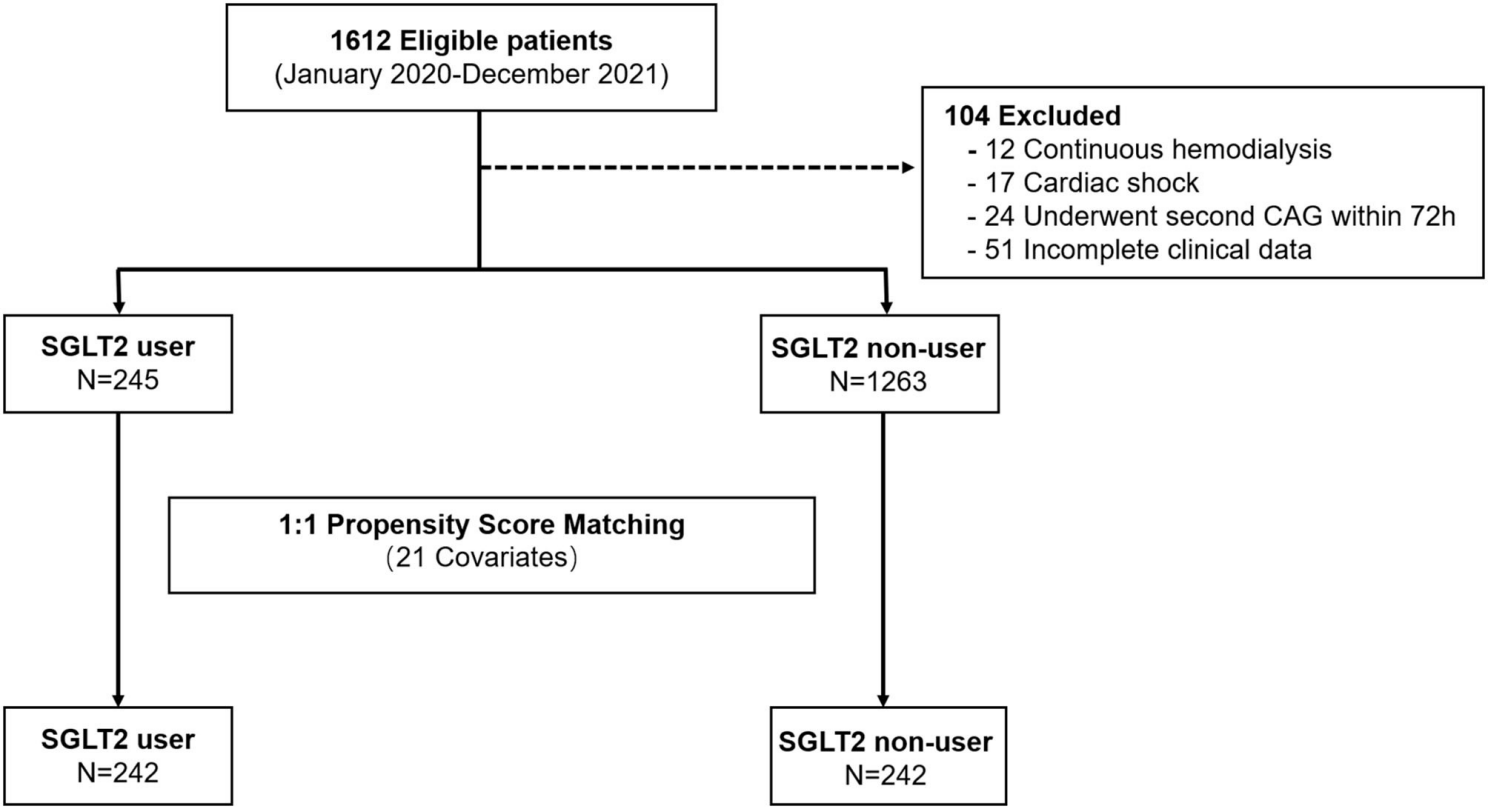
Study flow showing the derivation of unmatched and propensity score–matched patient cohorts in this study.

This single-center, retrospective, case-control study was approved by the institutional review board of the Medical School of Xi'an Jiaotong University and the need for informed or written consent was waived as part of the study approval. The patients were all admitted to the cardiology department of the First Affiliated Hospital of Xi'an Jiaotong University and underwent coronary angiography (CAG) and PCI between 1 January 2020 and 30 December 2021 (Figure 1). For this study, those patients with a diagnosis of T2DM and CAD and available serum creatinine measurements during hospitalization were included (*N* = 1,510). The diagnosis of T2DM was confirmed by fasting glucose (>7.0 mmol/L) or by a previous diagnosis when the patient was taking an oral hypoglycemic agent or insulin (9). CAD was defined as the presence of at least 50% luminal diameter narrowing in at least one major coronary artery by two experienced interventional cardiologists (10).

### Patient and Public Involvement

The study involved no patient or public in the development of the research question, outcome measures, study design, recruitment, conduct, and result dissemination.

### Definition of Exposure

The exposure of interest was a prescription of an SGLT2 inhibitor, including canagliflozin, empagliflozin, or dapagliflozin, for at least 6 months till the date of PCI. This was determined by the provider's prescription in the electronic medical record. Six months were used as a cut-off value as SGLT2i may exhibit the beneficial effects after a 6-month period as indicated in several studies (11, 12). For SGLT2 inhibitor users, the index date was defined as the first date on which an SLGT2 prescription was ordered. For nonusers, the index date was defined using the creatinine measurement date from 2020 to 2021.

### Definition of Outcome

The primary outcome was the first AKI event during the hospitalization. We identified CI-AKI events using a laboratory-based algorithm, which identifies events based on European Society of Urogenital Radiology (ESUR) serum creatinine criteria (increase in serum creatinine by ≥44.2 μmol/L or 0.5 mg/dL within 72 h or increase in serum creatinine by ≥1.25 times baseline value; hereafter referred to as CI-AKI_ESUR_) (13). As a sensitivity analysis, we also ascertained inpatient episodes of AKI using Kidney Disease: Improving Global Outcomes (KDIGO) serum creatinine criteria (increase in serum creatinine by ≥26.52 μmol/L/0.3 mg/dl within 48 h or increase in serum creatinine by ≥1.5 times baseline value before CAG; hereafter referred to as AKI_KDIGO_) along with the dates (14). The last laboratory values before undergoing CAG were used as the baseline data for analysis to best reflect each patient's baseline condition. The renal functions (serum creatinine and urea nitrogen) of patients with CAD were collected and measured conducted by automatic biochemical immune analyzer VITROS5600 (JNJ, New Jersey, US) upon admission and at 24, 48, and 72 h after CAG. The eGFR (ml/min/1.73 m^2^) was calculated *via* the modification of diet in renal disease equation: 186.3 × (creatinine in mg/dl) – 1.154 × (age in years) – 0.203 × (0.742 if woman) × (1.21 if black).

### Propensity Matching

All new users of SGLT2 inhibitors included in this study were identified from the electronic medical record. After being admitted to the hospital, each patient enrolled in this study by prescription screening was further inquired carefully about their drug history by doctors before PCI. Patients indeed taking SGLT2 inhibitors were then included as described. The exclusion criteria were (1) patients with acute myocardial infarction and requiring emergency PCI; (2) severe heart failure, left ventricular ejection fraction ≤ 40%; (3) complex coronary lesions that require extended operation time, or those with other contrast agents more than 400 ml; (4) severe arrhythmia; (5) severe hepatic (serum alanine transaminase >3 times the upper normal limit) and renal dysfunction (serum creatinine >221 μmol/l) or liver dysfunction; (6) urinary infection; (7) patients with poor glycemic control [HbA1c (%) >8%, fasting blood glucose >8.3 mmol/L or postprandial blood glucose >13.3 mmol/L], for whom the immediate change of glycemic treatment approaches was needed; and (8) patients with obvious hypotension, shock, and other causes of renal insufficiency before or after surgery.

Control subjects were selected among patients who never received a prescription for SLGT2 inhibitors and who had a diagnosis of diabetes since January 1, 2020. The data were collected from the hospital information system and prescription records were accessed centrally. Meanwhile, the prescription history of patients was inquired about by physicians on the first day of admission and double-checked by another doctor before PCI. Propensity scores were calculated as described (15) using logistic regression of SLGT2 inhibitor use on age, sex, creatinine measurement, hypertension status, insulin use, antihypertensive medication use, nonsteroidal anti-inflammatory drug use, eGFR, and HbA1c, with matching on previous pharmacist visit, diabetes duration, previous AKI episode, ACE inhibitor/angiotensin receptor blocker use, and volume of administered contrast, generating a separate score for control subjects. For participants with missing values in any of these covariates, exact matches were required on missing status. Propensity matching was performed with a 1:1 match for case and control subjects in which the nearest neighbor was selected without replacement.

### Statistical Analysis

Quantitative variables were summarized using means and SDs while qualitative variables were summarized as absolute and percentage frequencies. All analyses were performed using SAS (version 9.4) software (SAS Institute, Inc.). Baseline characteristics were compared between patients using Student's *t*-test for near-normal continuous variables, the Mann–Whitney *U*-test for other continuous variables, and the chi-square test for categorical variables unless more than 20% of cells had expected frequencies <5 in which case Fisher's exact test was used. After propensity score matching, two groups were compared using McNemar's test to evaluate the presence of correlation between CI-AKI events and the use of SGLT2 inhibitor over nonusers. All *P* values were two-sided and *P* < 0.050 was considered statistically significant.

## Results

### Study Populations

We identified a total of 245 SGLT2 inhibitor users and 1,265 patients with T2DM not on SGLT2 inhibitors who underwent PCI before matching. Compared with nonusers, SGLT2 inhibitor users tended to be at a lower age, with significantly lower comorbidities, namely, heart failure and PCI history, compared with SGLT2 inhibitor nonusers. They also had higher eGFR and a higher proportion of metformin use compared with nonusers. These results are shown in Table 1.

**Table 1:** Basic characteristics of patients in two groups before propensity matching.

**Variables**	**User (*n* = 245)**	**Nonuser (*n* = 12,65)**	***P*-Value**
**Demographics**
Female	56 (22.8)	336 (26.56)	0.2332
Age	62.2 (55–62)	64.6 (58–72)	<0.01^**^
**Comorbidities**
Smoking^*a*1^	136 (55.5)	661 (52.3)	0.3640
NYHA grade ≥III	8 (3.3)	89 (7.0)	<0.01^**^
STEMI	23 (9.4)	156 (12.3)	0.2344
PCI	48 (19.6)	362 (28.6)	<0.01^**^
Hypertension	150 (61.2)	823 (65.1)	0.2511
CKD	29 (11.8)	191 (15.1)	0.0154^*^
**Laboratory variables**
eGFR (ml/min/1.73 m^2^)	96.5 (79.8–111.6)	88.7 (75.1–103.0)	<0.01^**^
HbA1c (%)	6.2 (5.4–6.5)	6.4 (5.5–7.0)	0.1279
Total cholesterol (mmol/L)	3.74 (3.02–4.27)	3.73 (3.04–4.28)	0.8534
**Physiologic variables**
BMI (kg/m^2^)	24.59 (21.1–28.6)	24.86 (21.6–28.9)	0.4510
SBP (mmHg)	135.7 (127–143)	136.3 (128–144)	0.8273
DBP(mmHg)	77.6 ± 11.2	77.3 ± 12.0	0.3907
**Medications**
Metformin	208 (84.9)	887 (70.1)	<0.01^**^
Insulin	159 (64.9)	840 (66.4)	0.4072
ACEI/ARB	132 (53.9)	724 (57.2)	0.3319
β-blocker	204 (83.4)	1,098 (86.8)	0.2985
CCB	51 (20.8)	268 (21.2)	0.8968
Diuretic	40 (16.3)	194 (15.3)	0.6949
Statins	208 (86.0)	1,112 (87.9)	0.1939
**Outcomes**
CI-AKI_ESUR_	13 (5.3)	121 (9.6)	<0.01^**^
CI-AKI_KDIGO_	10 (4.1)	101 (8.0)	0.03^*^

*Continuous variables are presented as median (IQR), whereas categorical variables are presented as n (%)*.

* *P < 0.05*,

** *P < 0.01*.

*^a1^Smoking status was considered positive if ever smoker*.

*BMI, body mass index; NYHA, New York Heart Association grades; STEMI, ST segment elevation myocardial infarction; PCI, Percutaneous coronary intervention; SBP, systolic blood pressure; SBP, diastolic blood pressure; CKD, chronic kidney disease; CCB, calcium channel blockers; ACEI/ARB, agiotensin converting enzyme inhibitors; ACE inhibitors/angiotensin receptor blocker; eGFR, estimated glomerular filtration rate; HbA1c, hemoglobin A1c; TC, total cholesterol*.

After propensity matching, we identified 242 SGLT2 inhibitor users and 242 nonusers. The majority of users were on dapagliflozin (71.1%), followed by empagliflozin (16.9%), and then canagliflozin (12.0%). Users and nonusers were well matched except for PCI history (19.8% vs. 12.0% in users vs. nonusers), HbA1c (6.9% vs. 6.4% in users vs. nonusers), and metformin usage (86.0% vs. 70.2% in users vs. nonusers) in the cohort (Table 2).

**Table 2:** Basic characteristics of patients in two groups in propensity-matched dataset.

**Variables**	**User (*n* = 242)**	**Nonuser (*n* = 242)**	***P*-Value**
**Demographics**
Female	37 (30.5)	36 (29.8)	1.00
Age	62.6 (55–63)	63.6 (57–71)	0.44
**Comorbidities**
Smoking status^*a*1^	136 (56.2)	124 (51.2)	0.27
Drinking history	24 (9.9)	36 (14.9)	0.13
NYHA grade ≥ III	8 (3.3)	14 (5.8)	0.28
STEMI history	23 (9.5)	34 (14.0)	0.16
PCI history	48 (19.8)	29 (12.0)	0.03^*^
Hypertension	148 (61.2)	161 (68.2)	0.23
CKD	28 (11.5)	35 (14.5)	0.42
**Laboratory variables**
eGFR (ml/min/1.73 m^2^)	96.6 (78.1–112.8)	98.4 (83.1–118.6)	0.08
BUN (mmol/L)	5.5 (4.5–6.9)	5.3 (4.3–6.1)	0.06
Scr (μmol/L)	70.1 (55.0–77.3)	66.9 (51.0–72.3)	0.17
HbA1c (%)	6.9 (6.1–7.2)	6.4 (5.6–6.6)	<0.01^**^
Fasting glucose (mmol/L)	7.4 (4.8–8.8)	7.1 (4.9–8.2)	0.24
TC (mmol/L)	3.9 (3.2–4.5)	4.0 (3.3–4.5)	0.49
**Physiologic variables**
BMI (kg/m^2^)	24.6 (22.1–26.7)	24.9 (22.3–27.0)	0.45
SBP (mmHg)	135.7 (127–144)	136.3 (128–145)	0.83
DBP (mmHg)	77.6 (70–84)	77.3 (70–85)	0.39
Contrast volume	149 (102–177)	141 (100–169)	0.20
**Medications**
Metformin	208 (86.0)	170 (70.2)	<0.01^**^
Insulin	159 (65.7)	160 (66.1)	0.92
ACEI/ARB	130 (53.7)	142 (58.7)	0.27
β-blocker	201 (83.1)	210 (86.8)	0.31
CCB	49 (20.2)	53 (21.9)	0.66
Diuretic	40 (16.5)	54 (22.3)	0.13
Statins	206 (85.1)	215 (88.8)	0.28
**SGLT2 inhibitor type**
Dapagliflozin	172 (71.1)	–	–
Empagliflozin	41 (16.9)	–	–
Canagliflozin	29 (12.0)	–	–

*Continuous variables are presented as median (IQR), whereas categorical variables are presented as n (%)*.

* *P < 0.05*,

** *P < 0.01*.

*^a1^Smoking status was considered positive if ever smoker*.

*BMI, body mass index; NYHA, New York Heart Association grades; STEMI, ST segment elevation myocardial infarction; PCI, percutaneous coronary intervention; SBP, systolic blood pressure; SBP, diastolic blood pressure; CKD, chronic kidney disease; CCB, calcium channel blockers; ACEI/ARB, agiotensin converting enzyme inhibitors; ACE inhibitors/angiotensin receptor blocker; eGFR, estimated glomerular filtration rate; HbA1c, hemoglobin A1c; TC, total cholesterol*.

### CI-AKI Events and Severity

The proportion of patients with CI-AKI events was 5.3% (13 out of 245) in the SGLT2 inhibitor user cohort and 121 out of 1,265 (9.6%) in the whole nonuser cohort (Table 1). After propensity matching, the proportion of patients with CI-AKI events in SGLT2 inhibitor users and nonusers was 4.9 and 11.6%, respectively (*P* < 0.01) (Table 3). Similar results were found using the KDIGO definition of AKI (4.1% in users vs. 9.1% in nonusers, *P* = 0.04). We then compared the severity of AKI events as defined by changes in creatinine from baseline and peak creatinine measures during an AKI event. Median changes in serum creatinine from baseline for the user and nonuser groups were 54.3 μmol/L [interquartile range (IQR): 32.8–74.5] and 78.7 μmol/L (IQR: 61.3–92.8; *P* < 0.01), respectively (Table 3). However, the difference in median peak creatinine measures in the user and nonuser groups was not significant (140.8 μmol/L, IQR: 105.5–159.0 vs. 162.5 μmol/L, IQR: 133.3–176.5, *P* = 0.12) (Table 3). Acute dialysis for CI-AKI occurred in one patient in the user group and two patients in the nonuser group.

**Table 3:** CI-AKI outcomes in the SGLT2 inhibitor user and nonuser groups in the propensity-matched cohorts.

	**User (*n* = 242)**	**Nonuser (*n* = 242)**	***P*-Value**
CI-AKI_ESUR_ (%)	12 (4.9)	28 (11.6)	<0.01**
CI-AKI_KDIGO_ (%)	10 (4.1)	22 (9.1)	0.04*
Peak Scr in CI-AKI_ESUR_ events	140.8 (105.5–159.0)	162.5 (133.3–176.5)	0.12
Change in Scr during CI-AKI_ESUR_ events	54.3 (32.8–74.5)	78.7 (61.3–92.8)	0.01*
Need for acute dialysis	1 (0.4)	2 (0.8)	1

Continuous variables are presented as median (IQR), whereas categorical variables are presented as n (%). *P < 0.05, **P < 0.01.

**Figure 2:**
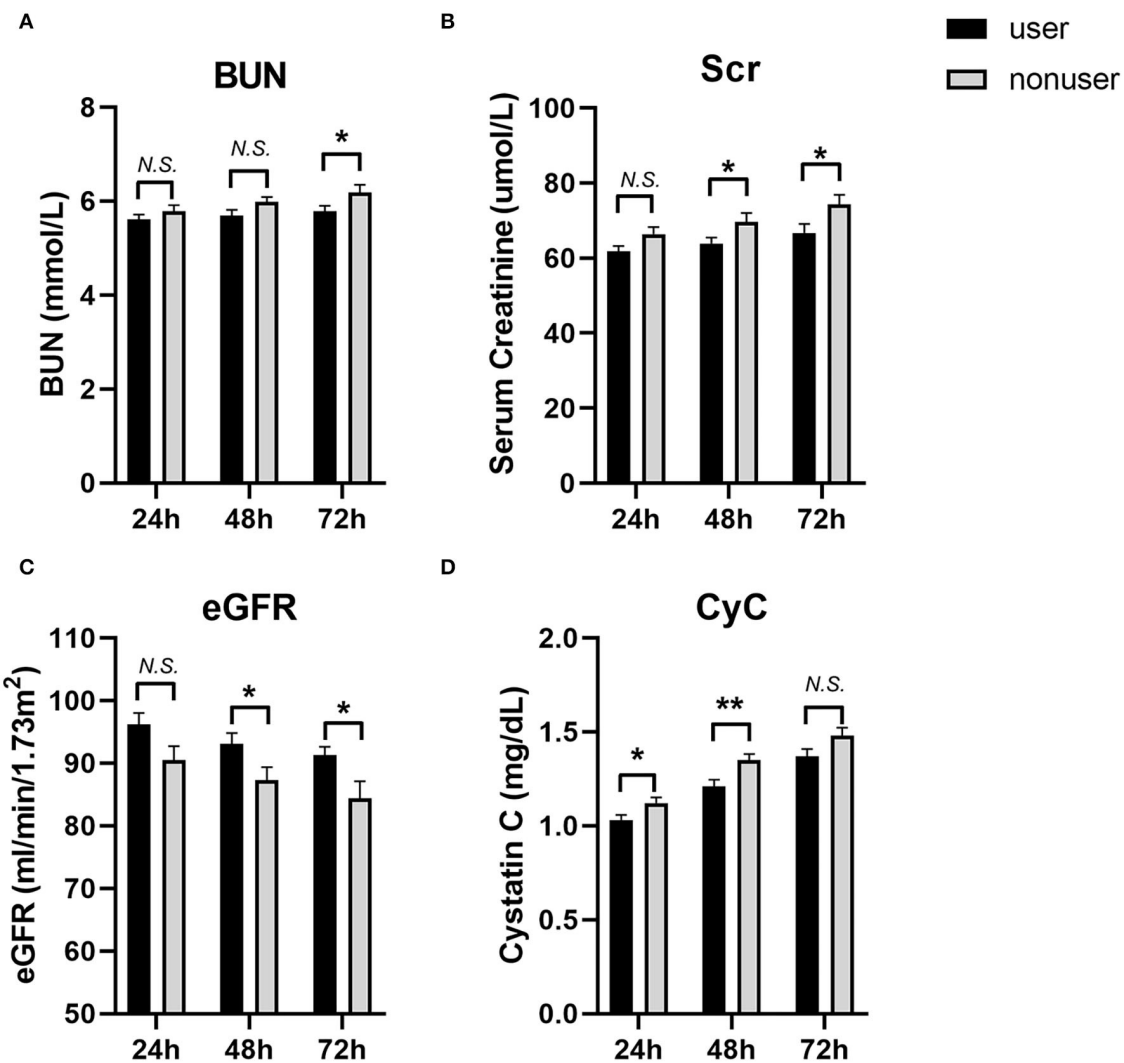
The levels of blood urea nitrogen (BUN), Serum creatinine (Scr), eGFR and Cystatin C (CyC) at 24, 48 and 72 h post-PCI. **(A)** BUN; **(B)** Scr; **(C)** eGFR; **(D)** CyC. User group: Black bars, *N* = 242; Nonuser group: Gray bars, *N* = 242. Data were shown as Mean ± SEM. Student's *t*-test for variables were used. *P < 0.05; **P < 0.01, N.S, not significant.

We also compared the levels of eGFR, creatinine, and blood urea nitrogen (BUN) at 24, 48, and 72 h post-PCI (Figure 2, Table 4). The levels of BUN were only significantly different at 72 h (5.78 ± 1.97 vs.6.19 ± 2.47, *P* = 0.044). The eGFR of users at 48 h (93.14 ± 26.51 vs. 87.33 ± 32.12, *P* = 0.031) and 72 h (91.26 ± 21.38 vs. 84.39 ± 42.76, *P* = 0.026) after PCI was significantly higher than those in the nonuser group. Accordingly, the levels of creatinine in SGLT2 inhibitor users were lower than that of nonusers at 48 h (63.88 ± 24.55 vs. 69.71 ± 36.81, *P* = 0.041) and 72 h (66.70 ± 37.47 vs. 74.31 ± 40.50, *P* = 0.032). The levels of cystatin C (CyC) in users were also lower at 24 h (1.03 ± 0.42 vs. 1.12 ± 0.48, *P* = 0.029) and 48 h (1.21 ± 0.55 vs. 1.35 ± 0.51, *P* = 0.004) compared to the nonuser group, but not significantly different at 72 h post-PCI (1.37 ± 0.61 vs. 1.48 ± 0.66, *P* = 0.058).

**Table 4:** Comparison of renal function and incidence of CI-AKI between the two groups at 24, 48, and 72 h after surgery.

	**User (*n* = 242)**	**Nonuser (*n* = 242)**	***P*-Value**
**BUN (mmol/L)**			
24 h	5.61 ± 1.62	5.78 ± 2.15	0.326
48 h	5.69 ± 1.81	5.98 ± 1.73	0.072
72 h	5.78 ± 1.97	6.19 ± 2.47	0.044*
**Scr (μmol/L)**			
24 h	61.83 ± 21.45	66.35 ± 29.97	0.057
48 h	63.88 ± 24.55	69.71 ± 36.81	0.041*
72 h	66.70 ± 37.47	74.31 ± 40.50	0.032*
**eGFR (ml/min/1.73 m** ^**2**^ **)**			
24 h	96.17 ± 29.12	90.47 ± 35.43	0.054
48 h	93.14 ± 26.51	87.33 ± 32.12	0.031*
72 h	91.26 ± 21.38	84.39 ± 42.76	0.026*
**CyC (mg/L)**			
24 h	1.03 ± 0.42	1.12 ± 0.48	0.029*
48 h	1.21 ± 0.55	1.35 ± 0.51	0.004**
72 h	1.37 ± 0.61	1.48 ± 0.66	0.058

Variables are presented as median ± SD. *P < 0.01.

*BUN, blood urea nitrogen; Scr, serum creatine; eGFR, estimated glomerular filtration rate; CyC, cystatin C*.

### Association Between SGLT2 Inhibitor and CI-AKI

**Figure 3:**
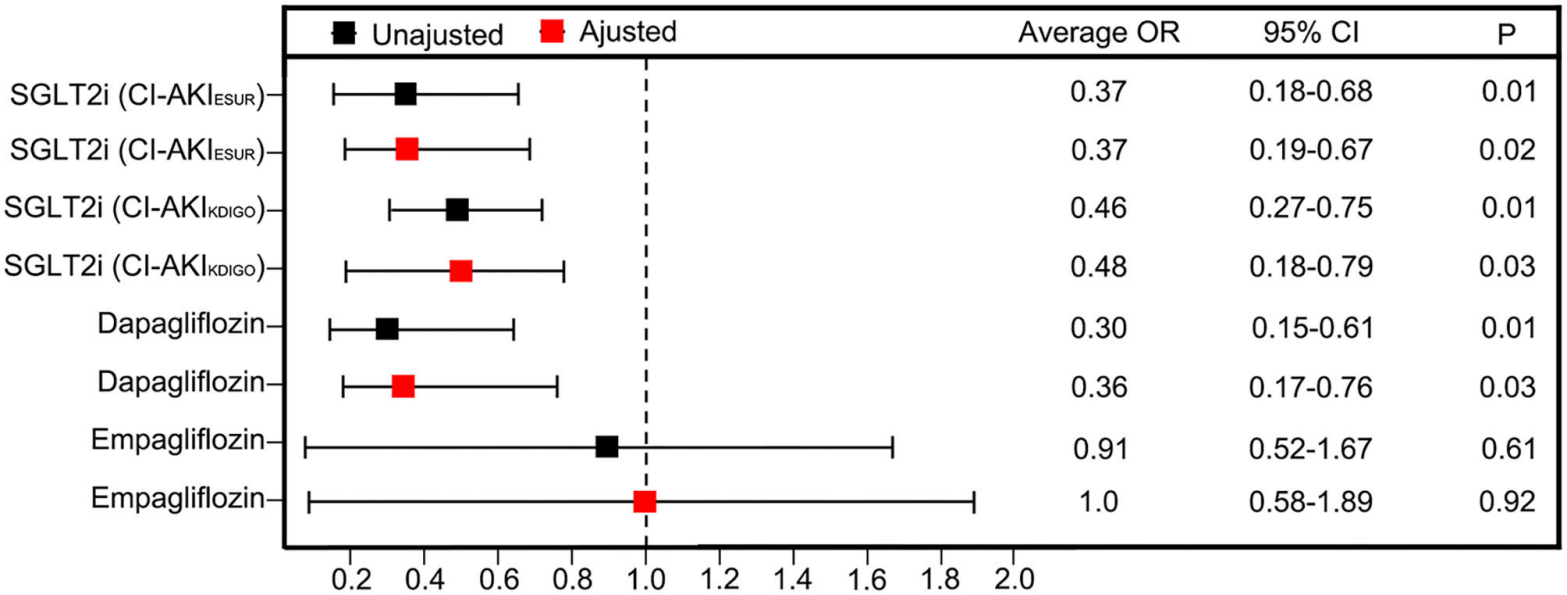
Unadjusted ORs and aORs of CI-AKI with 95% CIs in the propensity-matched cohorts. The ORs are generated after adjustment for covariates including age, sex, PCI histroy, NYHA grade ≥ III, HbA1c and metformin use.

The unadjusted ORs of CI-AKI_ESUR_ were 63% lower in the SGLT2 inhibitor user group [0.37 (95% CI: 0.16–0.88); *P* = 0.01] compared with the nonuser group (Figure 3). Sensitivity analysis using KDIGO definition also rendered similar results [0.46 (95% CI: 0.276–0.75); *P* = 0.02]. After adjusting for age, sex, PCI history, New York Heart Association (NYHA) grade ≥III, HbA1c, and metformin use, the adjusted OR of CI-AKI_ESUR_ remained unchanged [0.37 (95% CI: 0.19–0.67); *P* = 0.02]. We also investigated whether or not there was the differential risk of CI-AKI associated specifically with canagliflozin or dapagliflozin. Results for canagliflozin were not available because of the small sample size and lack of model convergence. The unadjusted and adjusted point estimates were qualitatively similar to the overall results (Figure 3). Furthermore, multivariate logistic regression analysis (Table 5) showed that age (OR: 1.06, 95%CI: 1.02–1.11, *P* = 0.01), PCI history (OR: 7.84, 95% CI: 3.26–18.84, *P* = 0.01), and NYHA grade ≥III (OR: 7.92, 95% CI: 1.80–34.91, *P* < 0.01) are also independent risk factors of CI-AKI for patients undergoing PCI.

**Table 5:** Odds ratios of CI-AKI after PCI by univariate and multiple logistic regression analysis.

	**Univariate logistic regression analysis**			**Multiple logistic regression analysis**		
	**OR**	**95% CI**	***P*-Value**	**OR**	**95% CI**	***P*-Value**
Age	1.06	1.01–1.13	<0.01^**^	1.06	1.02–1.11	0.01^*^
Sex (female)	0.42	0.21–0.95	0.03^*^	0.48	0.22–1.06	0.07
PCI	8.71	3.46–22.13	<0.01^**^	7.84	3.26–18.84	0.0*1
NYHA ≥III	8.13	1.91–37.52	<0.01^**^	7.92	1.80–34.91	<0.01^*^
Baseline eGFR	1.31	1.14–2.32	0.03^*^	1.01	1.00–1.32	0.09
Baseline BUN	1.08	0.83–1.45	0.04^*^	1.06	0.91–1.27	0.39
Fasting glucose	1.06	0.94–1.12	<0.01^**^	1.03	0.92–1.11	0.50
HbA1c (%)	1.18	0.89–1.52	<0.01^**^	1.13	0.82–1.48	0.16
Diuretic usage	2.10	0.79–5.50	0.14	–	–	–
SGLT2i usage	0.37	0.18–0.68	0.01^*^	0.37	0.19–0.67	0.02^*^

*CI-AKI, contrast-induced acute kidney injury; PCI, percutaneous coronary intervention; NYHA, New York Heart Association grades; CI, confidence interval; eGFR, estimated glomerular filtration rate; BUN, blood urea nitrogen*.

* *P < 0.05*,

** *P < 0.01*.

## Discussion

In this large contemporary cohort with different commodity burdens, and differing levels of renal function, we did not observe any increased risk of CI-AKI with SGLT2 inhibitor usage after PCI, consistent with previous study regarding AKI incidence (15). In contrast, we observed trends toward decreased risk of CI-AKI with SGLT2 inhibitor use before and after propensity matching, the estimates of which were qualitatively similar across several sensitivity analyses. The incidence of CI-AKI in patients with CAD and diabetes was lower in the user group, and SGLT2 inhibitor usage was found to be an independent protective factor for the occurrence of CI-AKI for patients after PCI. Moreover, the severity was not worse in the user group when CI-AKI occurred, as estimated by peak serum creatinine or change in creatinine. Finally, there was no increased risk of CI-AKI specifically for dapagliflozin and empagliflozin, for which there is a concern of AKI, and alerts have been issued (6, 15, 16).

Contrast-induced acute kidney injury (CI-AKI) is a common complication of patients undergoing PCI, often caused by diagnostic and therapeutic procedures using iodinated contrast media. Even slight, transient contrast-induced renal damage can have a negative impact on short- and long-term cardiovascular and renal prognoses (13). The common risk factors of CI-AKI are reported to include traditional factors (age, chronic renal failure, heart failure, and diabetes) and factors related to intervention, such as the dosage and types of contrast agents, multiple operations in a short period of time, and the use of intra-aortic balloon counter pulsation (17). Similar to this in our study, results showed such factors as age, NYHA grade, and PCI history risk factors for CI-AKI after PCI. Meanwhile, the point estimates for risk of CI-AKI were qualitatively similar for all the three types of SGLT2 inhibitors, suggesting a class effect. We believe our data complement and build upon the clinical trial data by the implementation of laboratory-based data using both diagnostic criteria of ESUR and KDIGO to ascertain AKI events, before and after propensity matching and additional adjustment, that are free from the selection bias (15).

Besides the overall nephroprotective effect, the risk of AKI with SGLT2 inhibitors is recognized since their clinical use. The Food and Drug Administration reports, and commentaries from various authors, have warned and speculated about AKI associated with SGLT2 inhibitors, namely, dapagliflozin and canagliflozin (16). As SGLT2 inhibitors act through inhibiting tubular glucose reabsorption, which results in a glucose-lowering effect and glucosuria, it is not inconceivable that they might cause acute perturbations in renal function (4). The additional natriuretic effect of SGLT2 inhibition may predispose patients with CAD, particularly those on diuretics and renin-angiotensin-aldosterone system antagonists, to experience abrupt reductions in GFR (16). Recently, several studies have provided evidence that SGLT2 inhibitors do not increase the risk for AKI in patients diagnosed with T2DM or heart failure (15, 18). Some studies also suggest that initiation of an SGLT2 inhibitor was associated with a reduced risk of AKI compared with other glucose-lowering strategies (15, 19, 20). In the Canagliflozin Cardiovascular Assessment Study (5), there were no statistically significant differences in the number of renal-related adverse events vs. placebo (19.7 vs. 17.4/1,000 patient-years). In the DECLARE-TIMI 58 (Dapagliflozin Effect on Cardiovascular Events-Thrombolysis in Myocardial Infarction 58) study, compared with placebo, dapagliflozin is confirmed to reduce newly increasing or worsening renal disease by 24% (11). In a meta-analysis, including three randomized controlled trials with a total of 1,831 patients, results showed that initiation of SGLT2 inhibitors in patients did not increase the AKI [OR: 0.76; 95% CI: (0.50, 1.16)] (20). These results suggest the renal-protective effects of SGLT2 inhibitors for patients with cardiovascular diseases.

However, in terms of CI-AKI, few studies have provided evidence of the effects of SGLT2 inhibitors on the risk of AKI for CAD patients undergoing PCI. Our study showed that CI-AKI incidence is lower in SGLT2-inhibitor users, suggesting further potential protective effects of SGLT2 inhibitors on the renal function of patients preparing for PCI. The most likely mechanism leading to CI-AKI is a rapid and sustained reduction in renal plasma flow preferentially to the outer medulla (21). This ischemia is due to an imbalance between renal vasodilatory and vasoconstrictive factors. SGLT2 inhibitor caused natriuresis, glycosuria, and following diuretic effect (4). These effects may help fluid administration correct volume depletion and expand IV volume, which increase the clearance of CM, decrease the concentration of CM in the tubule lumen and vasa recta, and counteract the activation of neurohormonal systems that lead to medullary vasoconstriction (22). We also found that levels of serum markers indicating renal function, namely, BUN, creatinine, and CyC, differed in user and nonuser groups. Higher levels of serum creatinine and lower levels of eGFR were observed at 48 and 72 h post PCI in the user group, further suggesting a role of an SGLT2 inhibitor in preserving renal function. On the other hand, CyC levels of patients at 24 h in the user group were lower than that of the control group, as is an earlier marker of AKI, suggesting the protective effects of the SGLT2 inhibitor may exert throughout the peri-PCI period.

The protective effects of SGLT2i have been validated in multiple studies regarding the cardiovascular and renal outcomes in patients with T2DM. In particular, 6 months after randomization in DECLARE-TIMI 58 trial, the mean eGFR in the dapagliflozin group and that in the placebo group started to show differences (11). Besides, according to the (Effects of Empagliflozin on Cardiac Structure in Patients with Type 2 Diabetes) study, SGLT2 inhibition was associated with a significant reduction in left ventricular mass indexed to body surface area after 6 months in people with T2DM and CAD (12). These studies indicate that usage of SGLT2i for more than 6 months may render the beneficial effects of cardioprotection and renoprotection for patients with T2DM. Thus 6-month usage of SGLT2i was used as the determination of inclusion for participants in this study. The impact of a shorter time of SGLT2i usage on the incidence of CI-AKI should be evaluated in future studies.

Our findings need to be interpreted with consideration of some limitations. This is a retrospective study based on a moderately sized cohort emanating from a single-center, thus ascertainment bias because of the retrospective nature of data is a possibility. Plus, the limited number of enrolled patients does not allow the assessment of specific outcome measures and subgroup analysis. Larger cohorts and multicenter studies are necessary to further assess the prognostic impact of SGLT2 inhibitor on CI-AKI risk for patients with T2DM scheduled for cardiac angiography. Meanwhile, as the two groups of patients may differ systematically, the potential effect of SGLT2 on CI-AKI events could be due to considerations for the commencement of SLGT2 inhibitors. In such cases, the comparisons are only suitable for patients who are legitimate candidates for SLGT2 inhibitors.

As American Dental Association recommended (23), injectable glucagon-like peptide 1 (GLP-1) receptor agonists (RAs) are the preferred option for patients with poor glucose control. However, some studies and case reports suggest GLP-1 RAs treatment tended to result in a nonsignificant decrease in risk for AKI (24, 25). Thus, excluding these patients may help to reduce confounding bias generated by GLP-1 RAs therapy in further analysis. Future studies will be needed to address the impact of SGLT2 inhibitors CI-AKI on patients with poor glycemic control.

In conclusion, our findings suggest that the risk of CI-AKI in real-life SGLT2 inhibitor users tended to decrease compared with matched nonusers, for legitimate candidates for SGLT2 inhibitors scheduled for PCI. These data suggest that the usage of SGLT2 inhibitor may benefit patients scheduled for PCI in preventing CI-AKI. Our analyses also demonstrate that advanced pharmacoepidemiologic methods can be leveraged to evaluate the potential risks of new and emerging drug classes. In the meantime, we believe the fear of CI-AKI associated with SGLT2 inhibitor use can be tempered for patients with CAD. Withdraw of SGLT2 inhibitor should not be recommended before PCI, to avoid inappropriate discouragement of the use of this novel class of agents that otherwise appear to afford significant long-term cardiovascular and renal protection in patients with T2DM.

## Data Availability Statement

The raw data supporting the conclusions of this article will be made available by the authors, without undue reservation.

## Ethics Statement

The studies involving human participants were reviewed and approved by Institutional Review Board of Medical School of Xi'an Jiaotong University (No. 2021-1492). Written informed consent for participation was not required for this study in accordance with the national legislation and the institutional requirements.

## Author Contributions

ZY and TL designed this study. TL, ND, and HG contributed to the collection and collation of the data. YW contributed to the explanation of the results and the analysis of the data. RH and TL wrote and revised the manuscript. All authors have reviewed the manuscript and the final version was approved by all.

## Funding

This work was supported by the National Key R&D Program of China Grants (2021YFA1301200 and 2021YFA1301201), the Special Foundation for State Major Basic Research Program of China (No. 92049203), the Youth Project of the National Science Foundation of China (No. 82000474), the Natural Science Foundation of Shaanxi Province of China (No. 2020JM-383), the Innovative Talents Promotion Plan of Shaanxi Province of China (No. 2021KJXX-04), and funding of Xi'an Jiaotong University (No. xzy012019093).

## Conflict of Interest

The authors declare that the research was conducted in the absence of any commercial or financial relationships that could be construed as a potential conflict of interest.

## Publisher's Note

All claims expressed in this article are solely those of the authors and do not necessarily represent those of their affiliated organizations, or those of the publisher, the editors and the reviewers. Any product that may be evaluated in this article, or claim that may be made by its manufacturer, is not guaranteed or endorsed by the publisher.
